# Supplementary material for: Characterization of Exterior Parts for 3D-Printed Humanoid Robot Arm with Various Patterns and Thicknesses
Source: Polymers (Basel). 2024 Apr 4;16(7):988. doi: 10.3390/polym16070988 (PMC11013340; doi:10.3390/polym16070988)
Supplement: Supplementary file 1 [file polymers-16-00988-s001.zip › polymers-2911616-supplementary.pdf]

Supplementary

< List of Tables >

Table S1. Sample code and slicing of 3D printed forearm of solid pattern with three different thickness
Table S2. Sample code and slicing of 3D printed forearm of re-entrant pattern with three different thickness
Table S3. Sample code and slicing of 3D printed forearm of solid pattern with three different thickness
Table S4. Sample code and slicing of 3D printed forearm of re-entrant pattern with three different thickness
Table S5. Sample code and slicing of 3D printed upperarm of solid pattern with three different thickness
Table S6. Sample code and slicing of 3D printed upperarm of re-entrant pattern with three different thickness
Table S7. Sample code and slicing of 3D printed upperarm of solid pattern with three different thickness
Table S8. Sample code and slicing of 3D printed upperarm of re-entrant pattern with three different thickness

Table S1. Sample code and slicing of 3D printed forearm of solid pattern with three different thickness

Sample code	Slicing
	Top
**FA_SL1**	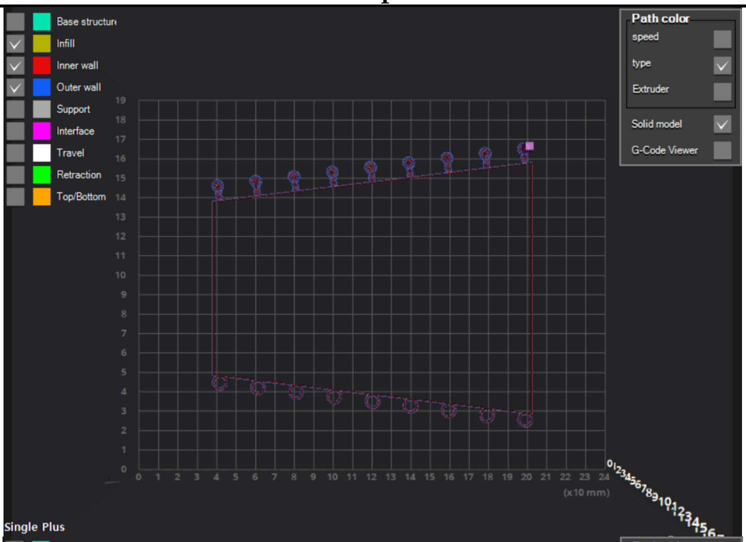
**FA_SL2**	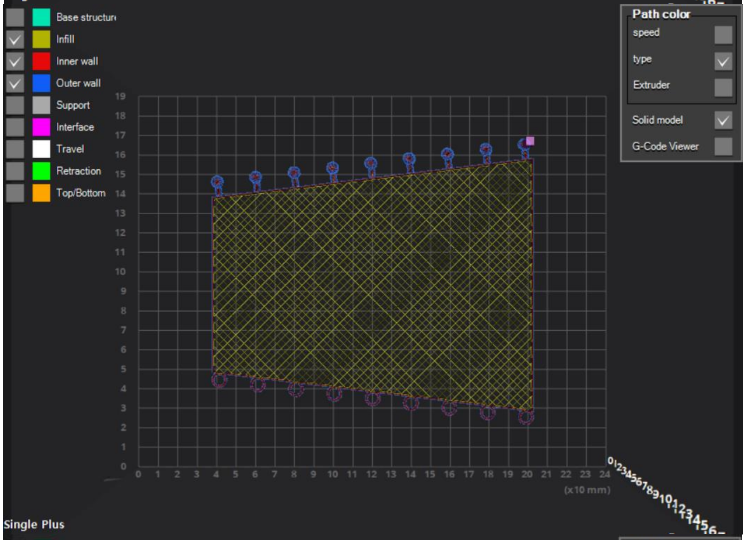
**FA_SL4**	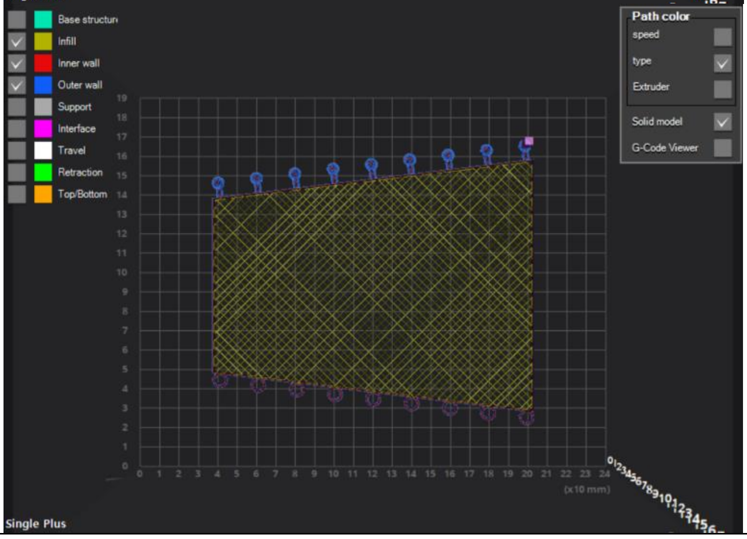

Table S2. Sample code and slicing of 3D printed forearm of re-entrant pattern with three different thickness

Sample code	Slicing
	Top
**FA_RE1**	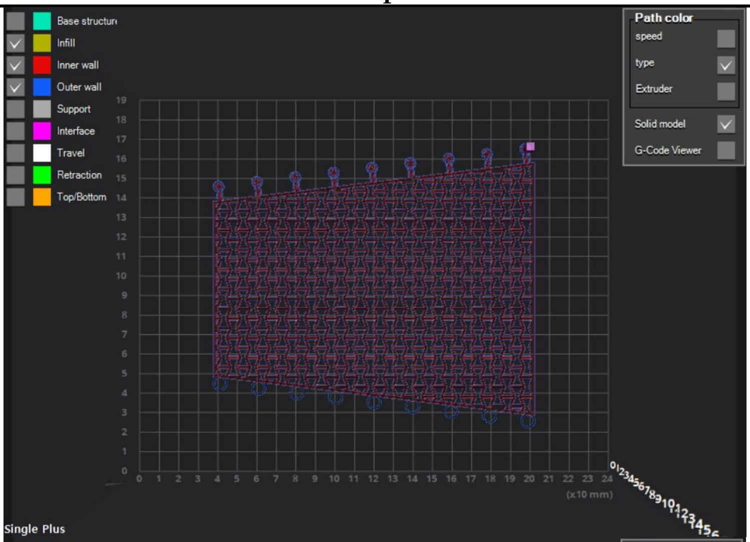
**FA_RE2**	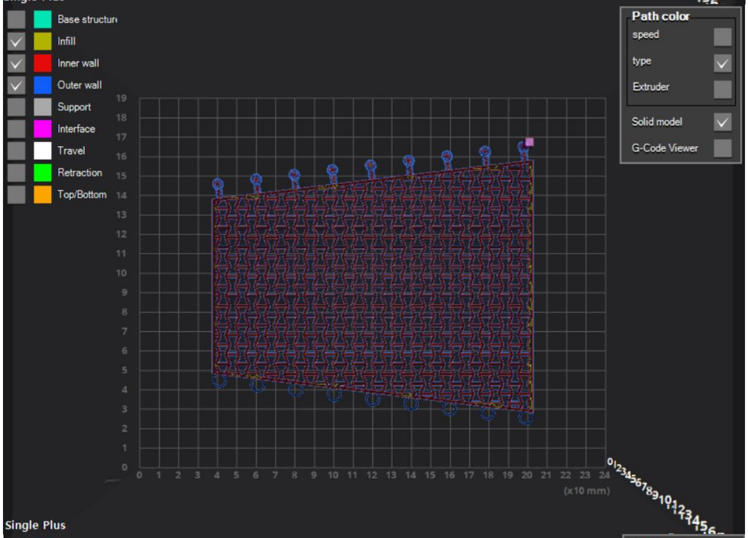
**FA_RE4**	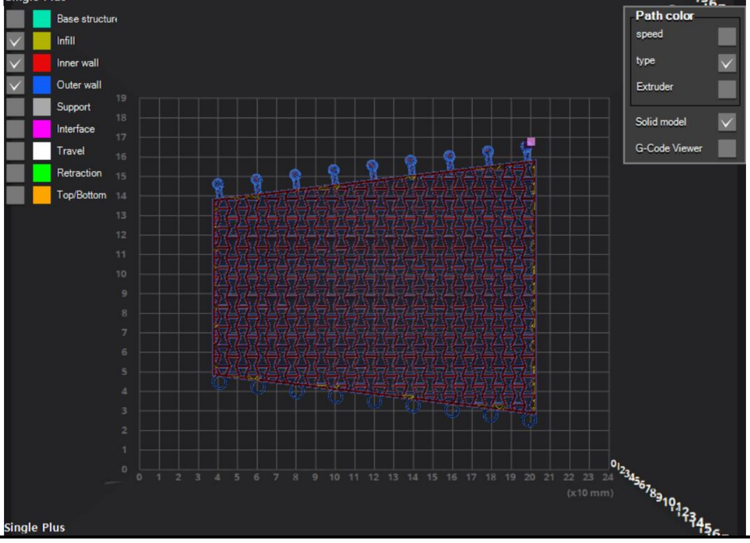

Table S3. Sample code and slicing of 3D printed forearm of solid pattern with three different thickness

Sample code	Slicing
	Front
FA_SL1	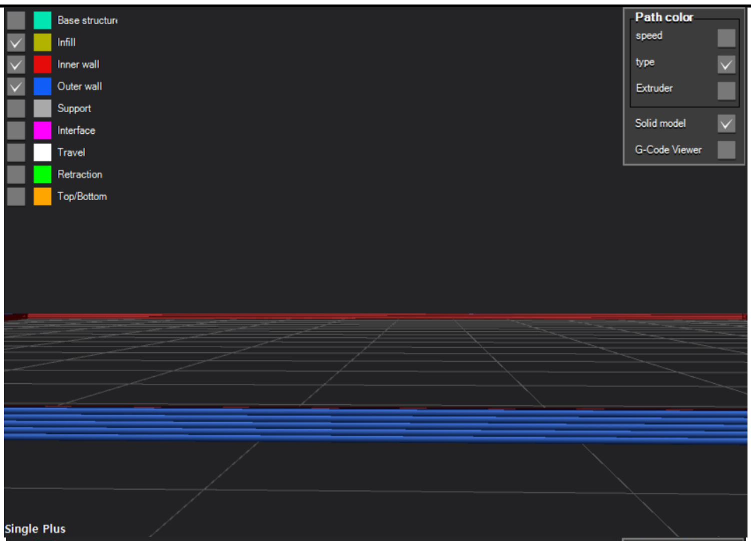
FA_SL2	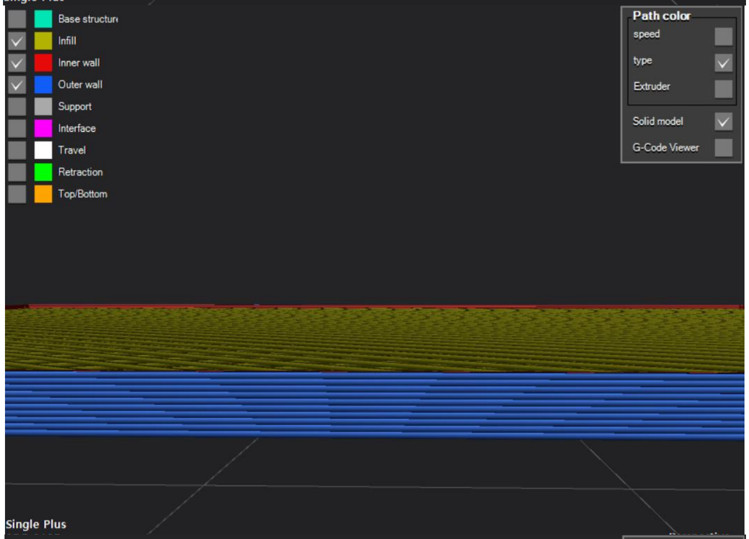
FA_SL4	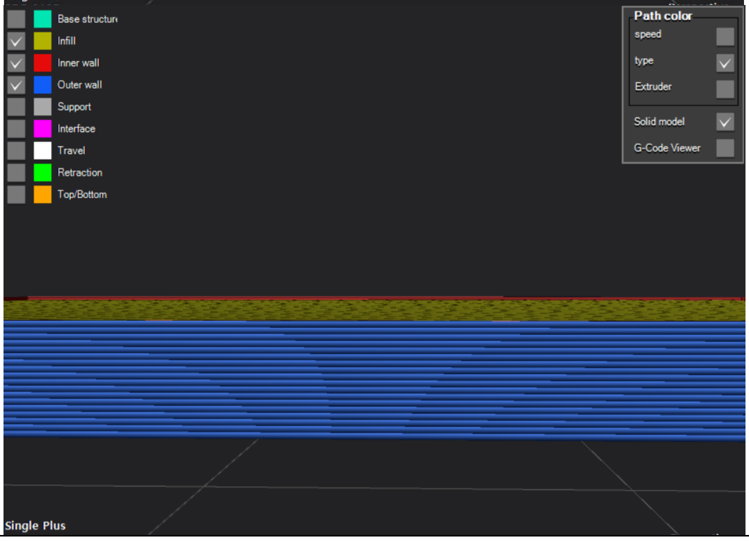

Table S4. Sample code and slicing of 3D printed forearm of re-entrant pattern with three different thickness

<table>
<tr><th rowspan="2">Sample code</th><th>Slicing</th></tr>
<tr><th>Front</th></tr>
<tr><td>FA_RE1</td><td>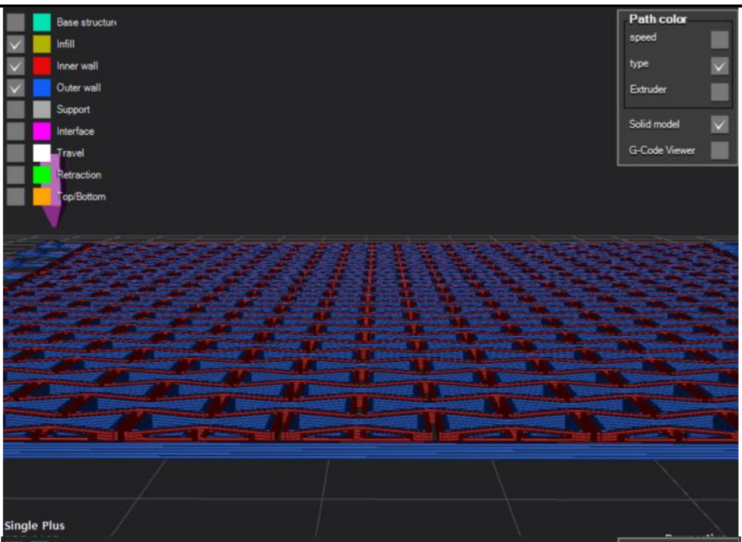
</td></tr>
<tr><td>FA_RE2</td><td>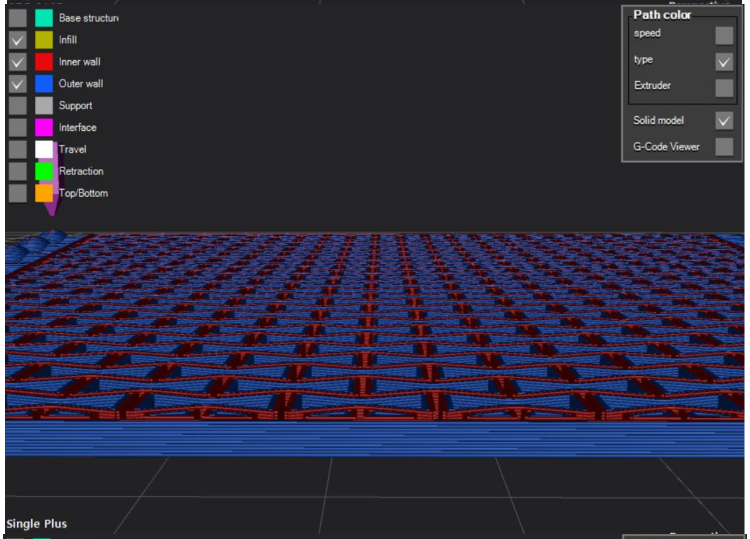
</td></tr>
<tr><td>FA_RE4</td><td>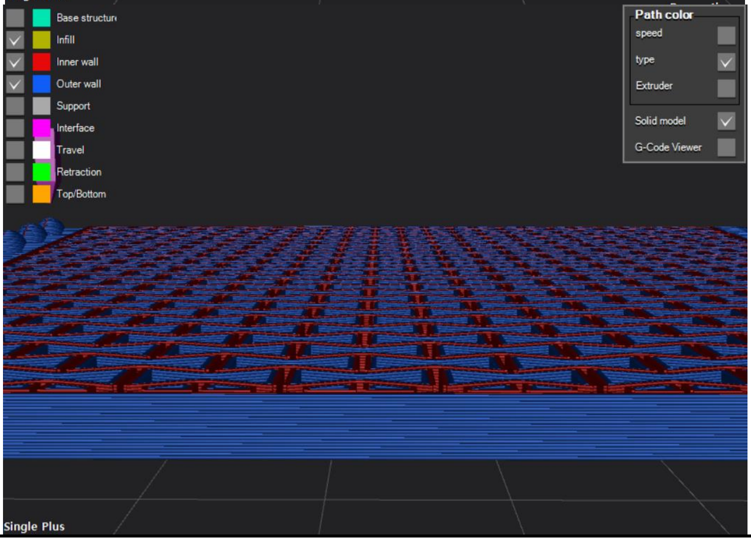
</td></tr>
</table>

Table S5. Sample code and slicing of 3D printed upperarm of solid pattern with three different thickness

Sample code	Slicing
	Top
UA_SL1	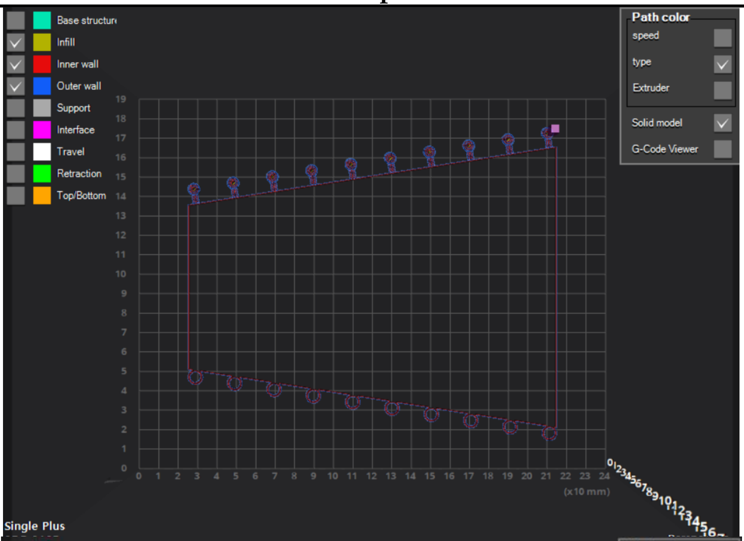
UA _SL2	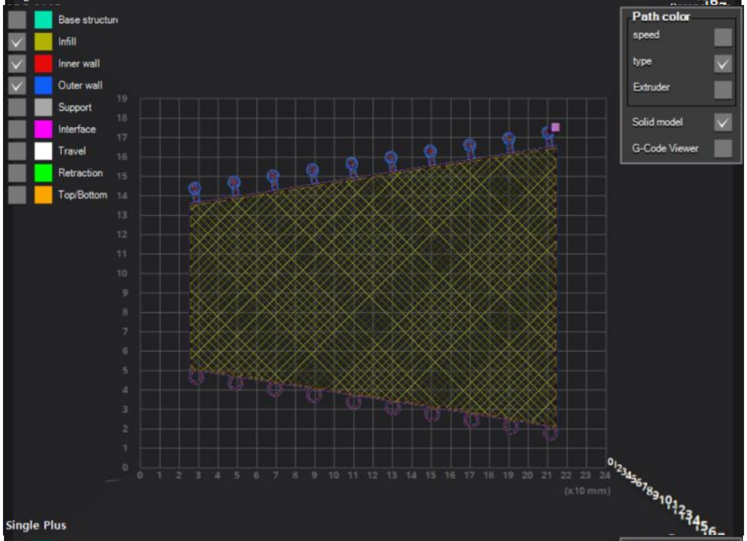
UA _SL4	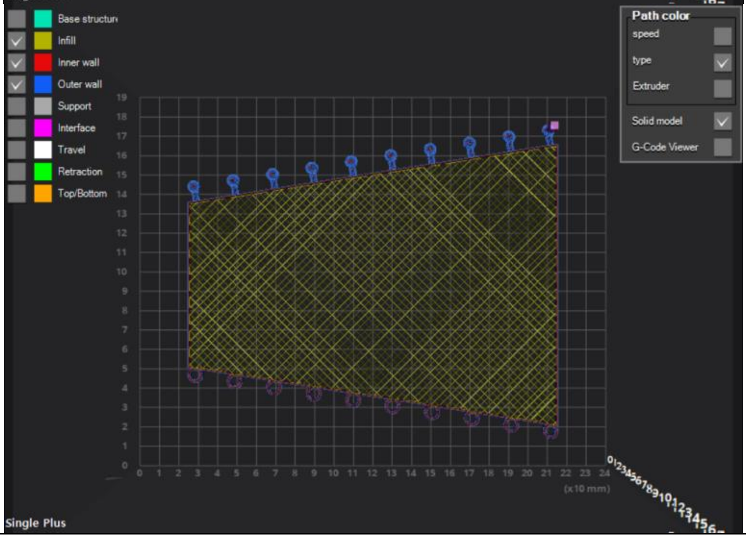

Table S6. Sample code and slicing of 3D printed upperarm of re-entrant pattern with three different thickness

Sample code	Slicing
	Top
UA _RE1	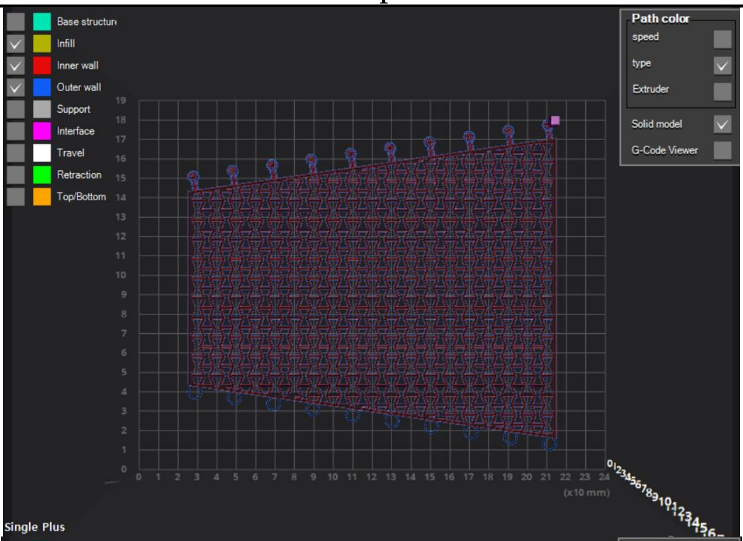
UA _RE2	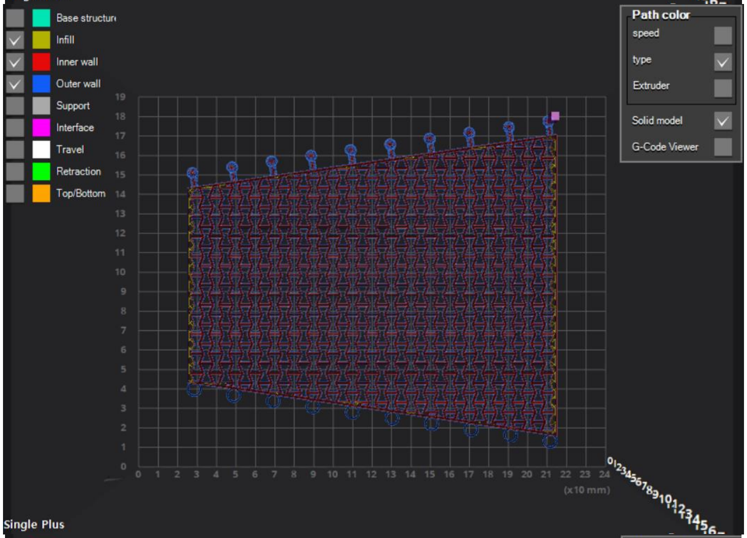
UA _RE4	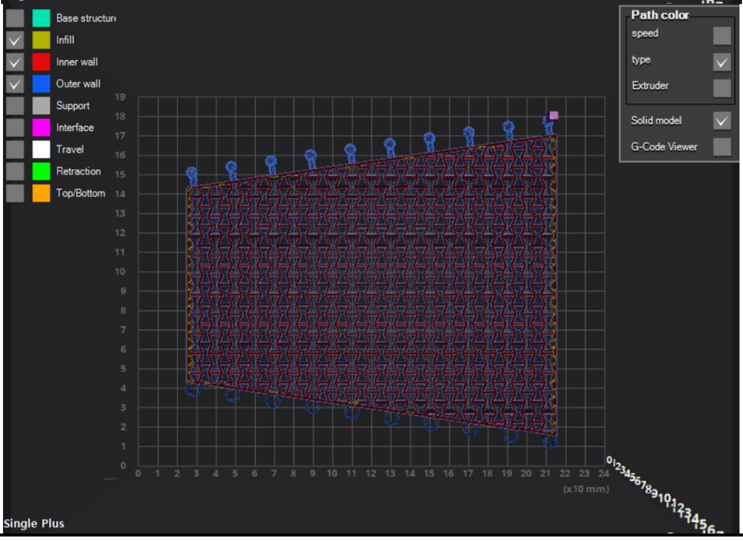

Table S7. Sample code and slicing of 3D printed upperarm of solid pattern with three different thickness

Sample code	Slicing
	Front
UA_SL1	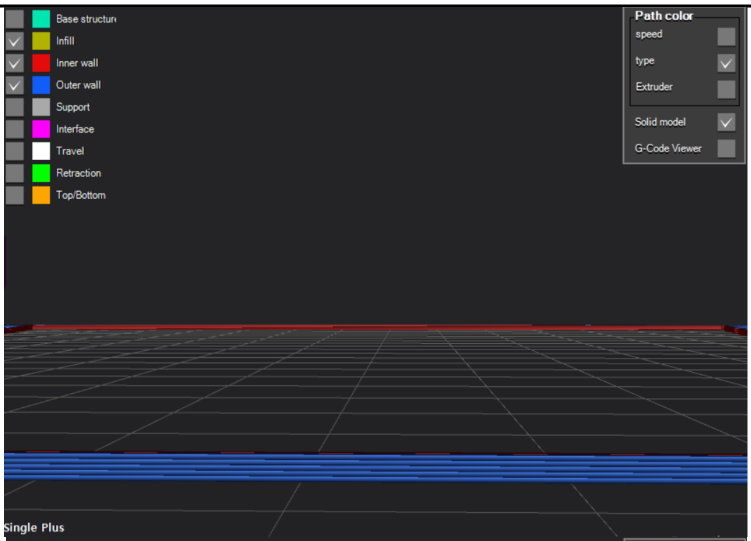
UA _SL2	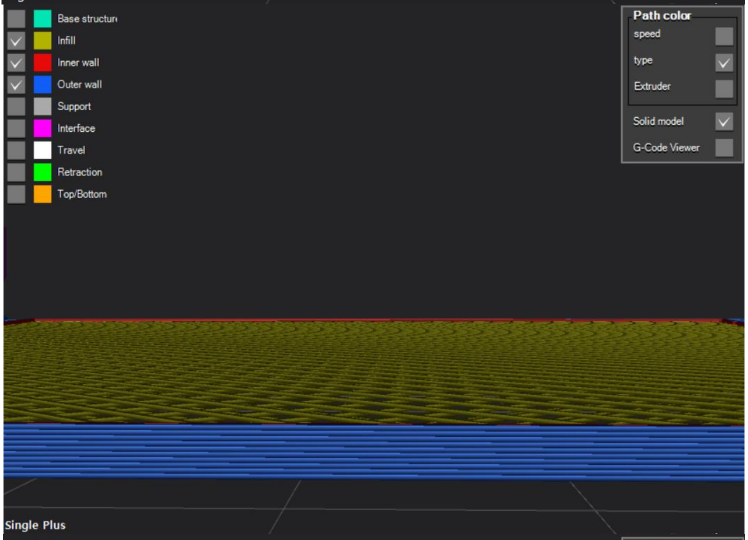
UA _SL4	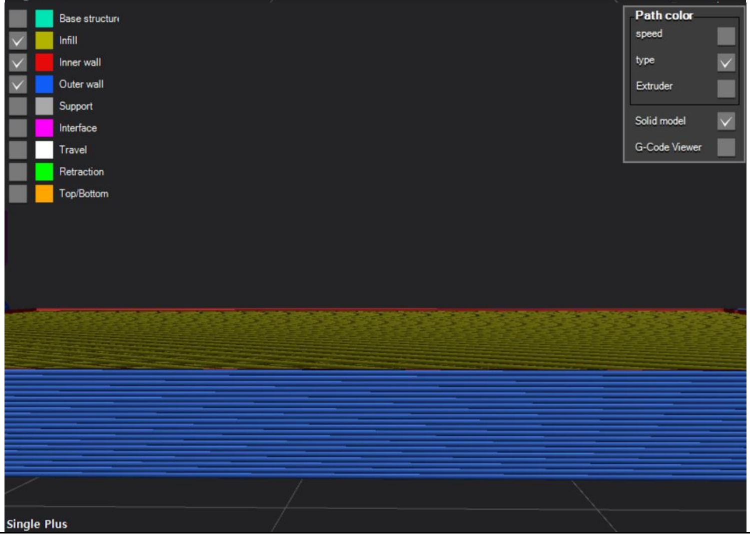

Table S8. Sample code and slicing of 3D printed upperarm of re-entrant pattern with three different thickness

Sample code	Slicing
	Front
UA _RE1	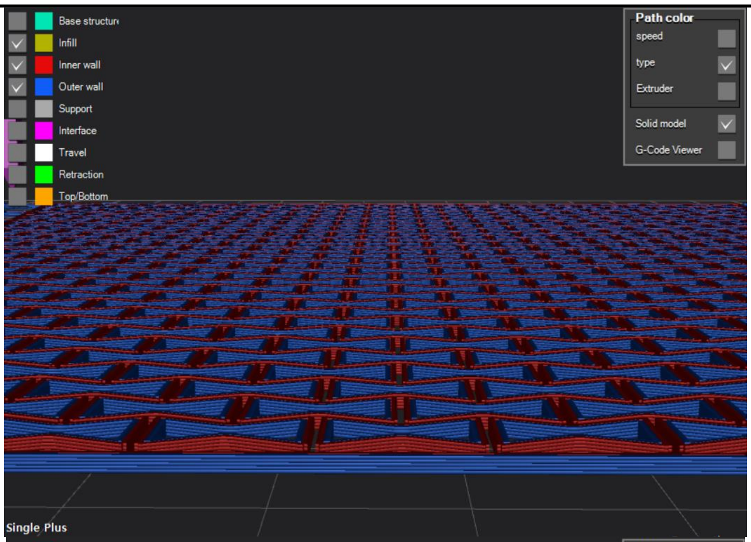
UA _RE2	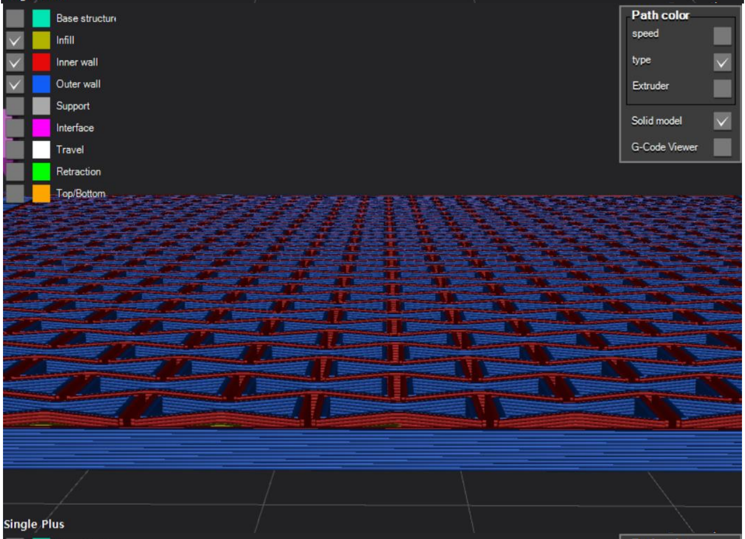
UA _RE4	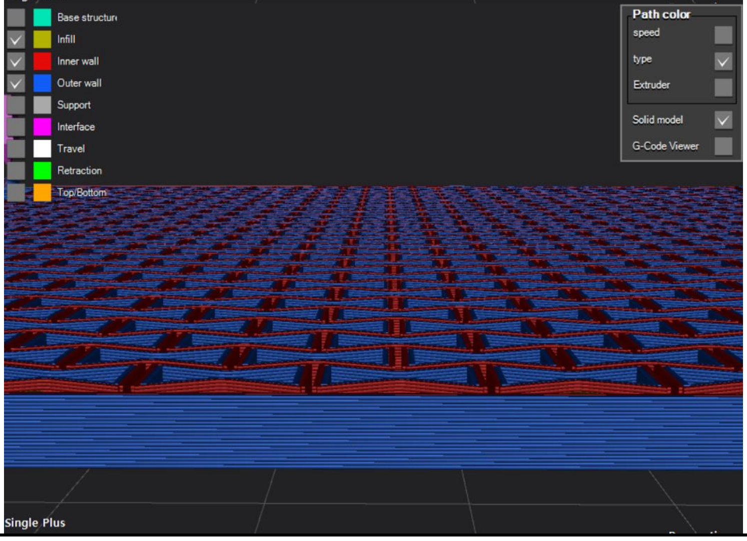
